# Perioperative mortality rate and its predictors after emergency laparatomy at Debre Markos comprehensive specialized hospital, Northwest Ethiopia: 2023: retrospective follow-up study

**DOI:** 10.1186/s12893-024-02401-7

**Published:** 2024-04-16

**Authors:** Megbar Dessalegn, Ayenew Negesse, Tilahun Deresse, Molla Yigzaw Birhanu, Eskeziyaw Agedew, Gedefaw Dires

**Affiliations:** 1 Department of Surgery, School of Medicine, Debre Markos University, Debre Markos, Ethiopia; 2 Department of Surgery, School of Medicine, Debre Birhan University, Debre Markos, Ethiopia; 3 Department of Public Health, College of Health Sciences, Debre Markos University, Debre Markos, Ethiopia; 4 Department of Human Nutrition, Health Science College, Debre Markos University, Debre markos, Ethiopia; 5 College of Health Sciences, Debre Markos University, Debre markos, Ethiopia

**Keywords:** Emergency laparatomy, Survival, Post-operative outcome, Mortality

## Abstract

**Background:**

Emergency laparatomy is abdominal surgery associated with a high rate of mortality. There are few reports on rates and predictors of postoperative mortality, whereas disease related or time specific studies are limited. Understanding the rate and predictors of mortality in the first 30 days (perioperative period) is important for evidence based decision and counseling of patients. This study aimed to estimate the perioperative mortality rate and its predictors after emergency laparatomy at Debre Markos Comprehensive Specialized Hospital, Northwest Ethiopia, 2023.

**Methods:**

This was a Hospital-based retrospective follow-up study conducted at Debre Markos Comprehensive Specialized Hospital in Ethiopia among patients who had undergone emergency laparatomy between January 1, 2019 and December 31, 2022. Sample of 418 emergency laparatomy patients selected with simple random sampling technique were studied. The data were extracted from March 15, 2023 to April 1, 2023 using a data extraction tool, cleaned, and entered into Epi-Data software version 3.1 before being exported to STATA software version 14.1 for analysis. Predictor variables with P value < 0.05 in multivariable Cox regression were reported.

**Results:**

Data of 386 study participants (92.3% complete charts) were analyzed. The median survival time was 18 days [IQR: (14, 29)]. The overall perioperative mortality rate in the cohort during the 2978 person-days of observations was 25.5 per 1000 person-days of follow-up [95% CI: (20.4, 30.9))]. Preoperative need for vasopressor [AHR: 1.8 (95% CI: (1.11, 2.98))], admission to intensive care unit [AHR: 2.0 (95% CI: (1.23, 3.49))], longer than three days of symptoms [AHR: 2.2 (95% CI: (1.15, 4.02))] and preoperative sepsis [AHR: 1.8 (95% CI: (1.05, 3.17))] were identified statistically significant predictors of perioperative mortality after emergency laparatomy.

**Conclusions:**

The perioperative mortality rate is high. Preoperative need for vasopressors, admission to intensive care unit, longer than three days of symptoms and preoperative sepsis were predictors of increased perioperative mortality rate.

## Background

Emergency laparatomy (EL) is a collective term for procedures to a variety of time-sensitive & urgent intra-abdominal surgical conditions that need surgical intervention shortly after the onset of symptoms [1, 2]. It accounts for 4.2 million deaths per year or 7.7% of all deaths [3]. These are 60% of procedures performed for emergency conditions [4, 5] in low-middle-income countries. Ethiopia has one of the lowest surgical volumes (148 per 100,000) [6]. However, emergency laparatomy is one of the ‘Bellwether procedures’ that can be affordable and accessible which is established by the Lancet Commission on Global Surgery [7].

Perioperative Mortality (POMR) is defined as in-hospital mortality due to any cause during surgery over the number of patients undergoing an operation. POMR is measured at two time periods: death on the day of surgery and before discharge from a hospital or within 30 days of the procedure, whichever is sooner [8, 9]. Thus, emergency laparotomies are time-sensitive abdominal surgeries associated with a high rate of mortality [2]. Although the estimation of POMR may be limited by the heterogeneity of definitions, the global incidence of postoperative mortality averages 4%. Despite limited reporting, perioperative mortality is twice higher in African settings [10–15].

In low and middle-income countries (LMICs), two-thirds of overall surgical procedures are performed for emergency conditions [4, 5]. Similarly, studies in Ethiopian teaching Hospitals showed that emergency laparatomy accounts for 23–36% of all surgical procedures performed [16, 17]. The related perioperative mortality rate is expected to be higher in poor countries than in high-income countries. As any surgery is inherently invasive, EL may result in postoperative complications including death [18]. At least 4.2 million people die worldwide within 30 days of surgery each year, and half of these deaths occur in LMICs making it the third greatest contributor to deaths, after ischaemic heart disease and stroke. This is higher than expected annual death from all-cause mortality related to HIV, malaria, and tuberculosis combined [19].

The disparity in perioperative mortality occurs in the presence of comparable postoperative complication rates reported from LMICs and high-income countries [12]. Emergency surgeries are expected to have three times mortality risk than planned surgeries [20]. The average mortality rate may reach up to 11.1% with a median length of stay equivalent to 16.3 days [21]. However, postoperative mortality risk is not evenly distributed across the postoperative period [22, 23]. Time bounded studies revealed an overall 30-day mortality of 17% [24]. A prospective study from Ethiopia revealed perioperative mortality incidence of 1.37 per 1000 person-day observations [25]. Another similar study to predict rates of mortality in the first 48 h postoperatively showed rates of 2.49% & 3.29% at 24 h and 48 h after surgery and anesthesia respectively with higher odds of mortality from emergency procedures [26].

A global target was set aiming that 80% of countries by 2020 and 100% of countries by 2030 will track perioperative mortality [19]. Although 18.2% of death in Ethiopia is from surgical causes, the Ethiopian national perioperative mortality rate (1.1% and 0.83% in 2019 & 2020 respectively) seems problematic from underreporting or difficulty in capturing the perioperative deaths [27, 28]. Moreover, studies on perioperative mortality in Ethiopia are limited to academic audits. Limited studies in Ethiopian teaching hospitals showed that emergency laparatomy account for 23–36% of all surgical procedures [16, 17]. However, these produced limited evidences on possible predictors of postoperative mortality and were inclusive of both elective and emergency conditions at a time [29].

Therefore, this study aimed to determine the mortality rate in the first 30-days (perioperative) and its predictors focused on the specific causes that need emergency laparatomy by including the time variable principles of survival analysis.

## Methods

### Study design

Hospital-based retrospective follow-up study was employed.

### Study area and period

The study was conducted at Debre Markos Comprehensive Specialized Hospital (DMCSH) in Debre Markos City, Northwest Ethiopia. Debre Markos City is located approximately 295 km northwest of Addis Ababa. It is a teaching hospital with 300 beds serving over five million people. It has 51 specialist physicians, 63 general practitioners, 386 nurses, and other support staff. The department of surgery is staffed with 15 surgeons and 18 general practitioners, 7 anesthetists to deliver elective and emergency surgical services. The surgical team is better organized after 2019. The emergency surgical service has a quarterly performance of 270 (75%) emergency procedures. The study was conducted from March 15, 2023 to April 1, 2023 on patients operated from January 1, 2019 to December 31, 2022.

### Population

#### Source population

Patients who had undergone emergency laparatomy at Debre Markos Comprehensive Specialized Hospital.

#### Study population

Patients who had undergone emergency laparatomy from January 1, 2019 - December 31, 2022 at Debre Markos Comprehensive Specialized Hospital.

### Inclusion and exclusion criteria

#### Inclusion criteria

All patients admitted and underwent emergency laparatomy between January 1, 2019 – December 31, 2022 at Debre Markos Comprehensive Specialized Hospital.

#### Exclusion criteria

All cases with simple appendectomy, cholecystectomy, trauma laparatomy, and obstetric laparatomy were excluded from the study as these patients have significantly different physiologic states. Charts of patients who were transferred from another Hospital after a surgical intervention or incomplete patient charts (without at least one progress note and discharge summary) were excluded from the study.

### Sample size and sampling procedure

Simple random sampling method was adopted as appropriate method to select a representative of emergency laparatomy patients based on identification number.

### Sample size determination

The total sample size was determined using a survival analysis formula [30] by assuming a one-to-one ratio of exposed to non-exposed, 95% level of confidence, and power of 80% and taking a mortality rate and Hazard Rate from the previous study in India [31]. The number of events (death) was calculated by applying the formula E = (Zα/2 + Zβ) ^2^ / (log (HR)) ^2^q0q1, where, z α/2 = 1.96, Z_β_ = 0.84, q1 = proportion of study participants participants that were in the exposed group and q0 = proportion of study participants particpants that were in the unexposed group, Hazard Ratio (HR) values of predictor variables from previous study and cumulative mortality rate (20.3%) from a previous study. After calculating the number of events (E), the optimum sample size (N) was calculated by dividing number of events with proportion of events (PE) using the formula (N) = E/PE, where PE is the [31]. Age as a post-emergency laparatomy mortality predictor yielded the largest sample size (380). The final sample size was determined to be 418 after adjustment by 10% for possible incomplete patient charts.

### Study variables

#### Dependent study variable

Perioperative mortality rate.

#### Independent study variables

**Patient socio-demographic factors**.

Age, sex, residence, mode of arrival, mode of admission, referral status.

**Preoperative factors** – Blood pressure, pulse rate, fever, abnormal leukocyte count, indication for surgery, duration of symptoms, presence of sepsis, presence of anemia, presence of comorbidity, use of prophylactic antibiotics, previous surgery, American Society of Anesthesiologists (ASA) status, vassopressor use, blood transfusion, diffuse abdominal tenderness, serum hemoglobin.

**Intraoperative variables** – Use of WHO checklist, duration of anesthesia, duration of surgery, blood transfusion, vasopressor use, bowel ischemia, degree of peritoneal contamination, source of peritoneal contamination.

**Post-operative variables** – Presence of postoperative complications, need for re-operation, Intensive care unit (ICU) admission, need for re-laparatomy, intra-abdominal collection.

### Operational definitions

Time: It is the number of days from the day of surgery to the occurrence of an event (death) or censoring.

Event: It is the occurrence of death within the first 30 days after emergency laparatomy.

Censored: Patients who underwent emergency laparatomy and were alive within 30 days, lost to follow-up, or transferred to another institution.

Incomplete patient charts: These were charts without at least one progress note and discharge summary.

Preoperative hypotension is blood pressure of less than 90/60 mmHg.

Abnormal leukocyte count is leukocyte count less than 4,000 or greater than 12,000.

### Data collection procedure and quality assurance

#### Data collection procedure and tools

This study used secondary data extracted based on a checklist prepared from literatures. It contained the following four sections; socio-demographic data, preoperative clinical data, intraoperative clinical data, and postoperative follow-up data. Data were collected by four trained nurses.

#### Data quality assurance

The data extraction checklist was evaluated by subject matter experts and checked on 5% of the sample for its applicability in extracting the necessary data. One day of training was given to the data collectors by the principal investigator before starting actual data collection. During the data collection period, close supervision and monitoring was conducted by the investigator.

### Data analysis

Data were entered using EpiData software version 3.1 and cleaning, coding, and analysis was done using STATA software version 14.1. Variance inflation factor pairwise comparison tests were performed to detect the presence of multicollinearity between independent variables. The Kaplan-Meier estimate was used to assess the survival experience of patients. A log-rank test was used to compare survival status between categorical variables.

Before fitting a regression model, proportional hazard assumption was checked using the Schoenfeld residual which was fulfilled in the global Schoenfeld residual test (calculated p-value = 0.81).

**Fig. 1:**
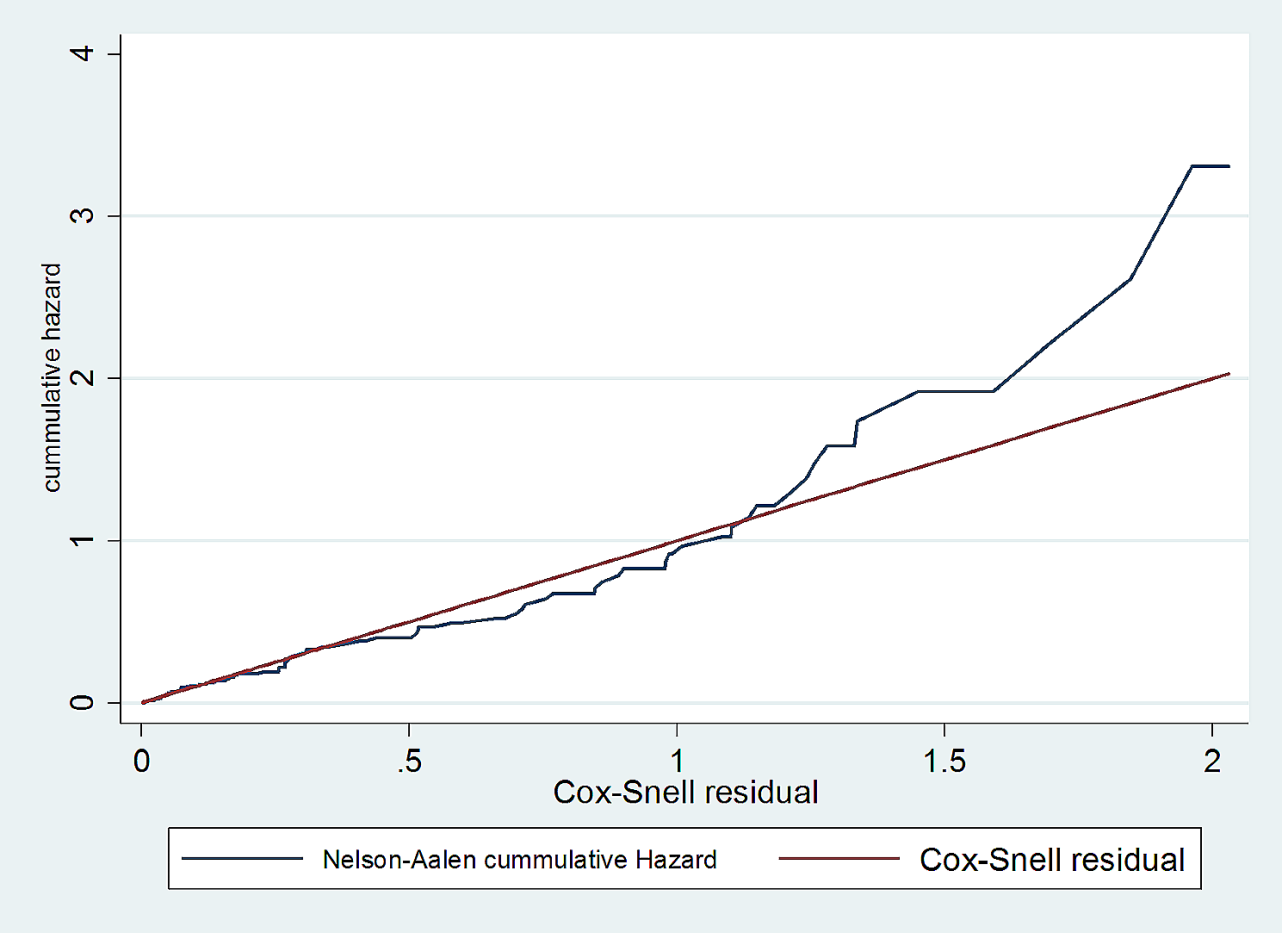
Cox-Snell residuals obtained by fitting Cox model for predictors of perioperative mortality, from January 1, 2019, to December 31, 2022

In the bivariable Cox regression analysis, crude hazard ratio (CHR) with a 95% CI was computed, and variables with a p-value < 0.25 were considered for multivariable analysis. In multivariable Cox regression analysis, the adjusted hazard ratio (AHR) with a 95% CI was computed, and a p-value < 0.05 was used to declare covariates as statistically significant predictors of perioperative mortality. Cox snell residual test was done for final model fit (Fig. 1).

Results were expressed as percentages, means with standard deviation, median with its interquartile ranges (IQR) and adjusted hazard ratio (AHR) along with its 95% confidence interval. Finally, the results were presented in text, tables and figures.

### Ethical consideration

Ethical clearance was obtained from the institutional review board (IRB) of Debre Markos University College of Medicine and Health Sciences. Subsequently, permission was obtained from the Debre Markos Comprehensive Specialized referral hospital’s quality assurance office, relevant departments, and unit heads of the hospital. There were no personal identifiers included from the patient’s medical record during data extraction, so it will not inflict any harm on the patients. All information used from the charts is kept confidential.

## Results

### Socio-demographic characteristics and medical condition of study participants

In this study, from the total sample, 386 charts of study particpants (92.3% complete charts) were included. The mean (standard deviation) age of participants at the time of admission was 38.0+17.9 years. The majority, (86.53%), of participants came from areas outside Debremarkos City Table 1 below.

**Table 1:** Socio-demographic characteristics of study participants, January 1, 2019, to December 31, 2022 (*N* = 386)

Covariates	Category	Event N (%)	Censored N (%)	Total N (%)
Age (years)	4–20	5(8.2)	56(91.8)	61(15.8)
	21–30	10(11.5)	77(88.5)	87(22.6)
	31–40	9(14.3)	54(85.7)	63(16.3)
	41–50	15(24.9)	43(74.1)	58(15.0)
	51–64	22(29.6)	50(69.4)	72(18.7)
	65–80	15(55.6)	20(44.4)	45(11.6)
Sex	Male	44(18.4)	195 (81.6)	239(61.9)
	Female	32(21.8)	115(78.2)	147(38.1)
Residence	Debre Markos	8(15.4)	44(84.6)	52(13.5)
	Out of Debre Markos	68(20.4)	266(79.6)	334(86.5)

### Mode of arrival and clinical characteristics of study participants

Most of the study participants, 314 (81.3%), were referred from other institutions and the median duration of symptoms was 3 days (IQR: (2–5)). Among these, nearly two-thirds (65.6%) arrived on the same day of referral. About 181(46.9%) of the study participants had conditions related to bowel obstruction. The majority of patients, (90.2%), were operated on the same day of admission. The median systolic and diastolic blood pressures at admission were 100 mmHg (IQR: (100–120)) and 70 mmHg (IQR: (60–70)), respectively (Table 3).

### Overall perioperative mortality rate after emergency laparatomy

In this study, there were 76 events. The incidence rate during the 2978 person-days of observations was 25.5 per 1000 [95% CI: (20.4, 30.9)]. The median (interquartile range) survival time for this study was 18, (14, 29) days. About seventy six (19.7%) of study participants had died during the study period while 301 (78%) were discharged improved, 4 (1.2%) left against medical advice, and five (1.2%) were transferred to other institutions.

The overall estimated survival rate after emergency laparatomy by the end of follow was 17.3% [95% CI: (5.00, 35.87%)]. The estimated cumulative survival was 98.4% [95% CI: (96.53, 99.29)] within the first 24 h of follow-up, and 97.3% [95% CI: (95.1, 98.6%)] after 3 days of follow-up. See Table 2 below.

**Table 2:** Log-rank test and median survival of patients in different groups, January 1, 2019, to December 31, 2022

No.	Variable	Value	Median survival time in days (95% CI)	*P* value
1.	Delayed presentation (> 3 days)	Yes		*P* < 0.001
		No	14 [95% CI: (12, 18)]	
2.	Need vasopressors	Yes	13 [95% CI: (10, 14)]	*p* < 0.001
		No		
3.	Immediate admission to ICU	Yes	12 [95% CI: (10, 21)]	*p* = 0.037
		No	24 [95% CI: (14, 30)]	
4.	Pus and/or gastro-intestinal content contamination	Yes	14 [95% CI: (12, 21)]	*p* < 0.001
		No	29 [95% CI: (18, 30)]	
5.	Duration of procedure	< 1 h	21 [95% CI: (14, 25)]	*P* < 0.05
		≥ 1 h	15 [95% CI: (13, 25)]	

**Fig. 2:**
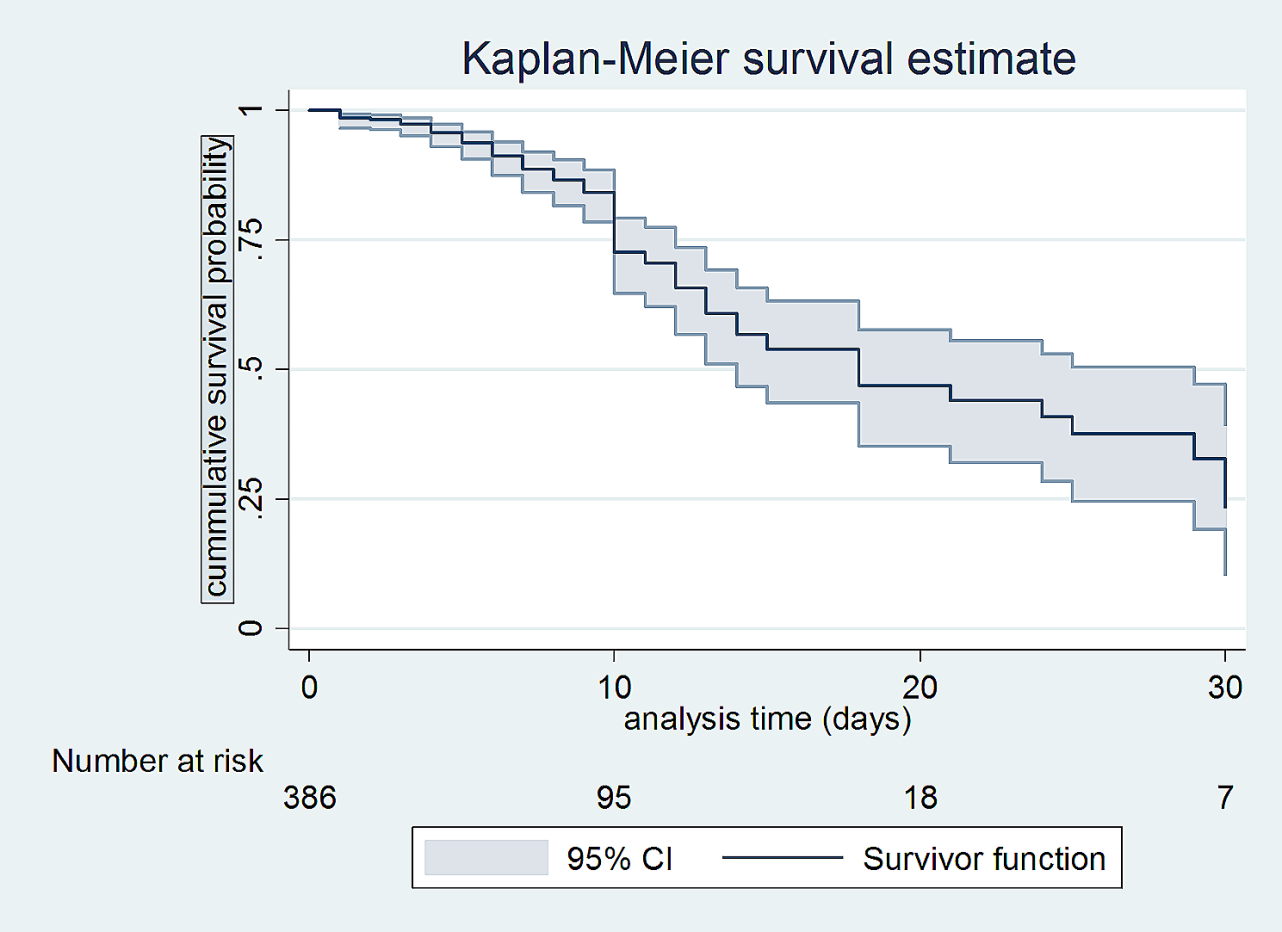
Estimated survival of patients after emergency laparatomy, January 1, 2019, to December 31, 2022

According to the survival curve for survival status after emergency laparatomy, the probability of survival rapidly drops between days 3 & 14 after emergency laparatomy (Fig. 2).

### Predictors of perioperative mortality after emergency laparatomy

In the bivariate analysis, duration of symptoms greater than 3 days, pus or fecal contamination of peritoneal cavity, longer operation time, preoperative vasopressor use, preoperative sepsis, degree of peritoneal contamination and immediate admission to intensive care unit were significantly associated with increased mortality after emergency laparatomy (*p* < 0.05). Abnormal leukocyte count, fever and bowel ischemia had p-value less than 0.25 and were included in the multivariable Cox regression analysis.

**Fig. 3:**
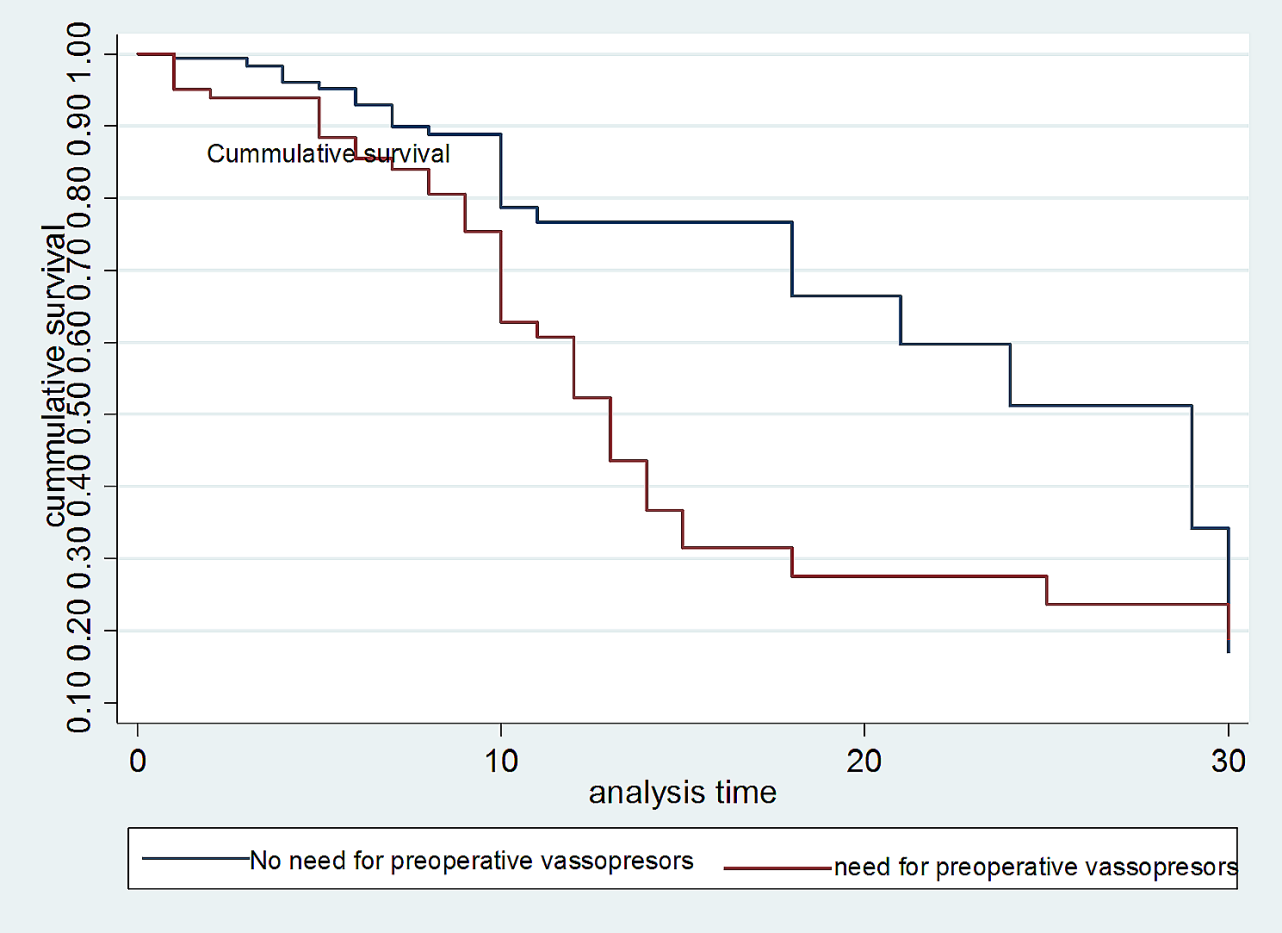
Kaplan Meier curves related to need for preoperative vasopressors, January 1, 2019, to December 31, 2022 (*N* = 386) Test of assumptions of Cox proportional hazards test

In the multivariable cox regression, preoperative vasopressor use and those with preoperative sepsis had 80% increased risk of death compared with patients who did not require it or had no preoperative sepsis (Fig. 3). The hazard rate of death among patients who presented after 3 days of symptoms was 2.2 times higher compared to those who presented earlier [AHR: 2.2 (95% CI: (1.2, 4.0))]. Patients who were transferred and cared in the intensive care unit (ICU) had twice [AHR: 2 (95% CI: (1.23, 3.49)] the risk of mortality compared to patients who were in the post-anesthesia recovery unit (Table 4, 5).

## Discussion

The purpose of this study was to determine the perioperative mortality status and its predictors after emergency laparatomy within the first 30 days of follow-up.

In this study, the perioperative mortality rate was 25.5 per 1000 person-days [95% CI: (20.4, 30.9)] in 2978 person-days of observation. These findings are higher than expected relative to previous national estimation in Ethiopia(0.83%) [28] and previous perioperative mortality studies in Ethiopia [25] which might be explained by the severity of the illness. The findings from this study are also higher than findings from a multinational prospective study done by the Global surgery collaborative group (14.2%) [13] and a Denmark study (17%) [24]. This difference might be explained by relative longer duration of symptoms which is a strong predictor of mortality in this study. These discrepancies may be further related to the relatively better quality of surgical care delivery and systems of care. Moreover, this rate is higher than the finding from a study at Dessie Referral Hospital (18.2%) [27] in Ethiopia and the Ethiopian national perioperative mortality report (1.1%) [28]. The difference in the rate of mortality might be explained by the inclusion of elective cases in the reporting of overall mortalities which might have moderated the overall rate of mortality. Perioperative mortality is a key quality indicator that is associated with high level process indicators in health care settings [32]. Similarly, the factors related with postoperative mortality may be beyond individual patient-related parameters. It might be associated with conditions like hospital-related adverse events [33, 34]. Perioperative mortality appears to be neglected but it can support a transition to high quality health systems in low and middle income countries. This can be achieved by analyzing postoperative mortality to understand the disease burden by monitoring and use it as an entry point to explore and diagnose system failures, practical priority setting and quality improvement programmes [35]. Early postoperative deaths may be considered from non-beneficial surgery that should be either postponed or needed further optimization [36, 37]. However, in our study, majority of the deaths (events) occurred after 3 days of hospital stay postoperatively (between 3rd to 14th days). The results in this study suggest that postoperative deaths were observed among patients who should benefit from the intended surgical intervention. This indicates that there is a window for practical improvement. Thus, reduction of postoperative mortality needs detailed study of contributing factors at individual and system level.

In this study, duration of symptoms was one of the factors that increase perioperative mortality. Patients who had emergency laparatomy three days after initial clinical symptoms (longer duration of symptoms) had more than two times more risk to die compared with patients who presented earlier. This finding is consistent with other findings from Ethiopia [38]. Longer duration of symptoms is associated with postoperative complications from delayed intervention [39]. In a Danish cohort study, every one hour delay in admission decreases survival by 2.4% [40]. The reasons related with this delay may be related with long referral chains [41] or related to delayed individual health seeking behavior from social or economic reasons [42, 43] or poor overall access to surgical services which takes more than 28.4 h to access a specialized hospital in Ethiopia [44]. In this study, most, 314(81.3%), of the cases are referred or transferred from other health institutions. Therefore, the problem related to delayed presentation needs further characterization to improve early admission, understand causes of delay and improvement in referral chain, or surgical care delivery within reasonable distance.

Emergency laparatomy done for patients who are cared for in the intensive care unit (ICU) immediately after laparatomy were two times more likely to die compared with patients who were transferred to the post-anesthesia recovery unit. Most of the admissions in this study, (41 of 45), were with ASA status IE & IIE which contrasts with nationwide databasis in Japan [45]. Similar studies from Ethiopia and others [38, 46, 47] reported admission to intensive care units to be associated with higher mortality. However, in these studies, 23.8% of patients were admitted at any time to the ICU postoperatively and the 30-day mortality seen among ICU patients was 37.9% which is proportionally higher than found in this study (11.65 and 36.6% respectively). Partly, the clinical judgment and selective admission of patients to intensive care unit may explain the higher risk of mortality. The patients included in this study are all those transferred to ICU immediately after the procedure. The mortality risk is expected to be higher in cases of unexpected ICU admission [48, 49]. It is practical that patients with risk score > 10% shall be admitted to ICU [50]. In reality, ICU care is expected to improve outcomes after surgery and it is one of the cost effective means of improving both short- and long term outcomes [51].

In this study, patients who had preoperative sepsis or needed vassopressors had increased risk of mortality by 80%. These findings are in line with studies from Ethiopia [52] and the United States of America [53]. The perioperative management of blood pressure improves surgical outcome. A systematic review showed hypotension or a change in blood pressure from baseline to increase postoperative mortality. Hemodynamic instability increases the risk of death in the postoperative period [54]. . The important difference between patients undergoing emergency laparatomy and those undergoing elective intra-abdominal procedures is presentation of the former in a state of physiologic derangement [55]. Hemodynamic stabilization through prompt assessment, resuscitation with goal directed fluid therapy is one of the standards in emergency laparatomy quality improvement bundles [56]. This results hold implication for evaluating adequacy of preoperative resuscitation based on preoperative care guidelines and evidence based decision on necessity of surgical intervention among patients who had preoperative sepsis and required vassopressors.

However, this study had some limitations. First, we assessed the acute postoperative complications until 30 days after surgery only, while delayed postoperative complications could occur even up to three months after surgery. Secondly, since this is a single-center study, the external validity of the study may be limited.

## Conclusion

The perioperative mortality rate from this study (25.5 per 1000 person-days) was higher than similar studies (1.37 per 1000 person days) in Ethiopia implying that emergency procedures have a greater risk The findings from this study implied that patients who presented later than three days of onset of symptoms, hemodynamic instability (with sepsis and preoperative need for vasopressors) and admission to intensive care unit were at a greater risk of perioperative death.

However, this study relied mainly on the time of presentation to hospitals and did not look into causes of delay from patients’ perspectives. In addition, the study span is limited to the first 30 days postoperatively. This needs further research beyond 30-days and institution (health system related factors) for wider understanding and holistic care.

## Annex

See Tables 3, 4 and 5.

**Table 3:** Mode of arrival and characteristics of study participants, January 1, 2019- December 31, 2022 (*N* = 386)

Covariates	Category	Event N (%)	Censored N (%)	Total N (%)
Referral status	Referred from other health institution	60(18.1)	254(81.9)	310(80.3)
	Came directly without referral	16(26.3)	56(73.7)	76(19.7)
WHO checklist use	Yes	46(19.2)	194(80.8)	240(62.2)
	No	30(20.5)	116(79.5)	146(37.8)
Comorbidity	Yes	74(71.2)	30(28.8)	104(26.9)
	No	236(83.7)	46(16.3)	282(73.1)
Mode of arrival	By ambulance	18(21.7)	65(78.3)	83(21.5)
	By themselves	58(19.2)	245(80.8)	303(78.5)
Duration of symptoms	≤ 3 days	14(10.6)	118(89.4)	132(34.2)
	> 3days	62(26.4)	192(75.6)	254(65.8)
Causes of Admission	Complications of appendicitis	10(6.8)	136(93.2)	146(37.8)
	Small bowel related	21(23.9)	67(76.1)	88(62.2)
	Large bowel related	29(31.2)	64(68.8)	93(24.1)
	Perforated peptic ulcer and Gall Bladder	16(27.1)	43(72.4)	59(72.9)
Preoperative hypotension	Yes	9(37.5)	15(62.5)	24(6.2)
	No	67(18.5)	295(81.5)	362(93.8)
Fever	Yes	26(27.4)	69(72.6)	95(24.6)
	No	50(17.2)	241(82.8)	291(75.4)
History of Previous Surgery	Yes	20(26.7)	55(73.3)	75(19.4)
	No	56(18.1)	255(81.9)	311(80.6)
Degree of Peritonitis	Localized	4(3.4)	114(96.6)	118(30.6)
	Generalized	72(26.9)	196(73.1)	268(69.4)
Severe anemia	Yes	74(19.4)	307(80.6)	381(98.7)
	No	2(40)	3(60)	5(1.3)
American Society of Anesthesiology status score	IE or IIE	69(18.5)	303(81.5)	372(96.3)
	IIIE or IVE	7(50)	7(50)	14(3.7)
Duration of Procedure	< 1 h	15(7.3)	191(92.7)	206(53.3)
	≥ 1 h	61(33.9)	119(66.1)	180(46.4)
Postoperative Complications	Yes	16(8.8)	166(91.2)	182(47.2)
	No	60(29.4)	144(70.6)	204(52.8)

**Table 4:** Results of bivariable and multivariable Cox proportional hazards regression analyses, January 1, 2019, to December 31, 2022 (*N* = 386)

Characteristics	Category	Event	Censored	CHR (95% CI)	AHR (95% CI)
Sex	Male	44	195	0.9 [0.6, 1.5]	1.1 [0.70, 1.99]
	Female	32	115	1	1
Duration of symptoms	> 3days	62	192	2.9 [1.3, 4.3]*	2.2 [1.15, 4.02]*
	≤ 3days	14	118	1	1
Presence of comorbidity	Yes	30	74	1.14 [0.72, 1.82]	0.8 [0.49, 1.38]
	No	46	236	1	
Abdominal tenderness	No tenderness	20	183	1	
	Diffuse tenderness	56	127	1.5 [0.89, 2.55]	0.72 [0.20–4.03]
Fever	Yes	26	69	1.4 [0.89, 2.32]**	1.5 [0.89, 2.53]
	No	50	241	1	1
abnormal leukocyte count	Yes	35	169	0.7 [0.46, 1.14]**	0.9 [0.49, 1.50]
	Normal	41	141	1	1
Diagnosis	Large bowel obstruction	64	288	1.0 [0.6–1.7]	1.0 [0.55, 1.81]
	viscus perforation	12	22	1.6 [0.9, 3.2]	1.2 [0.58, 2.92]
Preoperative antibiotic use	Yes	18	145	1.4 [0.8, 2.4]	1
	No	58	165		0.9 [0.66, 2.10]
Preoperative vasopressor use	Yes	41	41	2.2 [1.4, 3.5] *	1.8 [1.11, 2.98] *
	No	35	269	1	1
Preoperative hypotension	Yes	9	15	1.8 [0.9, 3.6]	1.1 [0.54, 2.51]
	No	67	295	1	1
Preoperative sepsis	Yes	29	37	1.8 [1.1, 2.9]*	1.8 [1.05, 3.17]*
	No	47	273		1
Degree of peritoneal contamination	Generalized	14	122	3.5 [1.24, 9.6]*	3.0 [0.96, 9.50]
	Localized or none	62	188	1	
Pus or faecal matter in peritoneal cavity	Yes	51	157	1.9 [1.2,3.0]*	1.7 [0.91, 2.93]
	No	25	153	1	1
Duration of procedures	≥ 1 h	61	119	1.96 [1.1, 3.5]*	1.7 [0.82, 3.40]
	< 1 h	15	191	1	1
Bowel ischemia	Yes	57	129	1.4 [0.81, 2.40]**	1.1 [0.54, 2.23]
	No	19	181	1	1
Site of Postop care	ICU	20	25	1.96 [1.2, 3.3] *	2.0 [1.23, 3.49]*
	PACU	56	285	1	1

*Note*: CI: confidence interval; AHR: adjusted hazard ratio; CHR: crude hazardratio; ICU: intensive care unit; PACU: postoperative anesthesia care unit

*p-value < 0.05;

**p-value < 0.25

**Table 5:** Test of proportional hazard assumption (Schoenfeld residuals) for variables, January 1, 2019, to December 31, 2022

Characteristics	rho	Chi2		Df		*P*-value
Duration of symptoms	0.16	1.81		1		0.19
Presence of comorbidity	0.08	0.49		1		0.48
Abdominal tenderness	-0.07	0.39		1		0.53
Fever	0.06	0.31		1		0.58
abnormal leukocyte count	0.05	0.29		1		0.59
Diagnosis	-0.07	0.51		1		0.48
Preoperative antibiotic use	0.05	0.29		1		0.59
Preoperative hypotension	-0.06		0.30		1	0.58
Preoperative sepsis	0.15		1.84		1	0.18
Degree of peritoneal contamination	0.65		0.35		1	0.55
Pus or faecal matter in peritoneal cavity	-0.10		1.09		1	0.29
Duration of procedures	0.008		0.01		1	0.93
Bowel ischemia	0.08		0.49		1	0.48
Site of Postop care	0.008		0.01		1	0.93
Global test			10.22		15	0.81

## Data Availability

The datasets used and/or analyzed during the current study is available from the corresponding author on reasonable request.

## Abbreviations

AHR: Adjusted Hazard Ratio
AOR: Adjusted Odds Ratio
ASA: American Society of Anesthesiology
CI: Confidence Interval
CHR: Crude Hazard Ratio
CT: Computed Tomography
DMCSH: Debre Markos Comprehensive Specialized Hospital
HIMS: Health Management Information System
HR: Hazard Ratio
ICU: Intensive Care Unit
LMICs: Low Middle-Income Countries
mmHg: Millimeter Mercury
OR: Odds Ratio
POMR: Perioperative Mortality Rate
WHO: World Health Organization
